# A Large Concept Model for Mechanistic Simulation of Disease Trajectories: A Hypothesis-Generating Exemplar for Pediatric Acute Lymphoblastic Leukemia

**DOI:** 10.34133/csbj.0154

**Published:** 2026-06-30

**Authors:** Wayne R. Danter

**Affiliations:** HumanQAI Inc. (formerly 123Genetix Inc.), London, Ontario, Canada.

## Abstract

**Background:** Many diseases evolve through complex, nonlinear trajectories shaped by interacting genetic, cellular, and environmental factors over time. Such dynamics are difficult to represent using static risk models, particularly in biologically heterogeneous conditions such as pediatric acute lymphoblastic leukemia (ALL). Here, we present a large concept model (LCM) as a mechanistic, hypothesis-generating framework for simulating longitudinal disease trajectories using pediatric ALL relapse dynamics as a proof-of-concept exemplar. **Methods:** We developed a causal longitudinal modeling framework implemented within the aiHumanoid v11.0 platform to characterize post-remission relapse dynamics. Seven clinically relevant ETV6::RUNX1-based genotypic profiles were simulated from remission baseline (T0) through 2 post-remission intervals (T1 = 3 months; T2 = 6 months). Longitudinal remission-to-relapse changes were evaluated across genotype- and age-defined virtual cohorts using descriptive nonparametric effect-size-oriented measures. Relapse dynamics were summarized using 2 composite system-level metrics: the relapse risk score and relapse pressure index. **Results:** The model generated distinct genotype- and age-associated trajectory patterns across relapse-relevant biological domains and produced composite measures reflecting modeled relapse pressure within the simulation environment. Greater relapse-associated biological divergence was observed in selected genotype–age strata, particularly in domains related to clonal evolution, treatment resistance, and minimal residual disease. **Conclusions:** This ALL-focused proof of concept demonstrates the architectural and analytic potential of mechanistic trajectory simulation for hypothesis generation, longitudinal systems modeling, and future integration with real-world longitudinal datasets.

## Introduction

The objective of the present study is to introduce and demonstrate a causal, mechanistic longitudinal simulation framework for characterizing post-remission relapse dynamics as a time-dependent biological process. Pediatric acute lymphoblastic leukemia (ALL) is used as an exemplar system to illustrate how genotype-specific and age-modulated relapse trajectories can be explored within a controlled computational environment. Using longitudinal simulation from remission baseline (T0) through 2 post-remission intervals (T1 and T2), the framework evaluates how relapse-relevant biological domains evolve over time rather than inferring risk from static measurements. This approach is intended to support methodological development in longitudinal disease trajectory modeling and hypothesis generation, rather than to provide direct clinical prediction or patient-level decision support. Throughout this manuscript, “trajectory” is used to denote the longitudinal evolution of simulated system behavior and state transitions within the aiHumanoid causal framework, summarized using discrete observation intervals rather than continuous biological measurements.

ALL is the most common childhood malignancy, accounting for approximately 25% of pediatric cancers. Since the earliest demonstrations of chemotherapy-induced remission in childhood leukemia [1–3], successive advances in risk-adapted, protocol-driven therapy have yielded contemporary remission rates exceeding 90% [4]. Despite these gains, relapse remains the leading cause of ALL-related mortality [5–7]. Post-remission disease monitoring is therefore challenged by the dynamic and often subclinical nature of relapse biology, which frequently emerges without clear clinical warning during surveillance. This highlights the difficulty of characterizing relapse as a temporally evolving biological process rather than as a discrete clinical event [8,9].

Minimal residual disease (MRD) assessment is central to current ALL risk stratification strategies, but it remains an inherently time point-dependent measure with recognized limitations in capturing relapse dynamics [10,11]. Although MRD quantifies residual leukemic burden at specific surveillance intervals, it does not fully reflect underlying biological processes such as clonal evolution, immune modulation, and treatment resistance that may unfold longitudinally following remission [12]. As a result, relapse risk is often inferred from static measurements rather than modeled as a trajectory, limiting the ability of existing approaches to represent the temporal structure and biological momentum of post-remission disease progression.

In our previous reported study, “Investigating genetic and familial risks in childhood ALL: A longitudinal virtual study using aiHumanoid simulations of ETV6::RUNX1 gene fusion” [13], we demonstrated that mechanistic, physiology-informed simulation could reproduce biologically plausible early disease behavior, including precursor immune dysfunction and hematologic perturbations, within virtual pediatric cohorts. That work established the feasibility of using causal simulation to explore longitudinal disease evolution in ALL under controlled biological assumptions. Building on this foundation, the present study extends the same methodological framework beyond leukemogenesis to the post-remission phase, focusing on how relapse-relevant biological processes evolve over time within a longitudinal simulation environment.

Increasing evidence suggests that relapse risk in pediatric ALL is developmentally modulated, reflecting age-dependent interactions among immune reconstitution, bone marrow microenvironmental dynamics, and therapy responsiveness. Consequently, relapse vulnerability may emerge preferentially within specific developmental windows rather than remaining constant over time. Incorporating age as an active biological dimension is therefore essential for accurate post-remission risk stratification.

To characterize subtle but important changes in modeled relapse-relevant trajectory behavior, we employed a descriptive nonparametric framework incorporating the Cliff’s delta [14] effect size and the Hodges–Lehmann estimator (HLE) [15]. This approach summarizes the magnitude, directionality, and ordinal separation of within-model longitudinal changes without implying population-level inference. We further developed composite relapse indices to support rapid, quantitative risk classification for each genotype.

In this study, we do not aim to establish validated clinical prediction or disease-agnostic generalizability. Rather, we present pediatric ALL as a focused exemplar to test whether an LCM can represent longitudinal disease behavior in a mechanistically structured and analytically interpretable way. Our goals are 3-fold: (a) to demonstrate how an LCM can be organized to simulate remission-to-relapse trajectories in ALL, (b) to examine whether the model produces biologically plausible age- and genotype-associated trajectory contrasts, and (c) to evaluate whether such simulations can serve as a basis for future hypothesis generation and subsequent empirical validation. Accordingly, the present manuscript should be interpreted as a proof-of-concept modeling study rather than a clinically validated predictive system.

## Methods

This study presents a computational framework for analyzing disease trajectories using large concept models (LCMs), illustrated here through a pediatric ALL exemplar. In this framework, biological systems are represented as directed causal graphs in which nodes correspond to biological concepts and weighted edges encode causal influences between concepts. System states evolve through synchronous updates of concept values, allowing complex disease dynamics to emerge from the underlying causal structure. The framework enables simulation of disease progression across time and supports structured comparison of trajectory patterns between simulated cohorts.

In this project, we employed the aiHumanoid [13] v11 simulation system, an LCM (LCM)-based theoretical framework designed to model virtual pediatric physiology and disease progression under explicitly defined causal assumptions. The purpose of this work is to present a hypothesis-generating methodological framework for longitudinal, causal simulation of post-remission disease dynamics within a pediatric ALL exemplar, rather than to predict individual clinical outcomes or replace existing clinical risk stratification tools. In this study, “risk” refers to the relative burden of relapse-relevant biological processes as represented within the simulation, rather than to estimated probabilities of clinical relapse events.

### LCM formulation

The aiHumanoid v11 engine is implemented as an LCM: a high-dimensional causal network in which each concept represents a biologically meaningful or biologically interpretable process (for example, MRD, clonal evolution, or immune activation), and directed edges carry signed weights in the $\left[-1,1\right]\;$ range encoding the strength and polarity of causal influence between concepts. In aiHumanoid v11, this LCM architecture is a direct evolution of the fuzzy cognitive map (FCM)-based structure used in earlier system versions, retaining the signed, weighted causal-graph data structure while extending it to a larger set of biologically grounded concepts and richer concept-level semantics. Concept states are updated iteratively by applying a bounded monotone activation function to the weighted sum of their inputs, yielding longitudinal trajectories of system states over time. Causal relationships and edge weights were defined using a combination of literature-informed biological reasoning, expert-guided model construction, and internal consistency constraints enforced through sober second thought (SST) validation. Composite relapse indices such as the ALL_relapse risk score and relapse pressure index (RPI) are implemented as additional concepts that aggregate relapse-relevant inputs (MRD, clonal evolution, treatment resistance, immune modulation) into system-level summaries of the underlying LCM dynamics. Recent work on LCMs in AI and mechanistic concept-level biomedical simulation provides broader context for this modeling approach [16,17].

Because several concepts in the pediatric ALL LCM represent biologically aggregated or systems-level states rather than one-to-one molecular observables, key variables were mapped to their intended biological interpretation, model role, and closest empirical analogue. In this study, concepts such as MRD, clonal evolution, pre-leukemic clone expansion, immune facilitation/inhibition, and the relapse composite indices were treated as biologically interpretable model constructs whose function may be mechanistic, proxy-like, integrative, or outcome-oriented depending on network position. Table S3 provides this interpretability mapping to clarify how these concepts relate to known pediatric ALL biology without implying direct equivalence to a single laboratory assay or clinical endpoint.

### Update rule and activation function

Within the aiHumanoid v11 LCM, all concept activation states are updated synchronously at each discrete iteration according to a common network-level update rule. For each concept, the model first forms a weighted sum of its causal inputs from the current iteration and then adds a small persistence term proportional to its own state from the previous iteration (t−1), introducing inertia into the dynamics.

Concept states were updated synchronously according to:

$$X_{i}\left(t+1\right)=f\left(\;\Sigma_{j}\;w_{\mathrm{ji}}\;X_{j}\left(t\right)+\alpha \;X_{i}\left(t\right)\right)$$ (1)

where *X_i_*(*t*) denotes the state of concept *i* at iteration *t*; *w_ji_* denotes the signed and weighted, causal influence of concept *j* on concept *i*; α denotes the persistence term; and *f*(·) denotes a bounded monotone activation function that maps updated concept states back to the system-standard range. This intermediate value is then passed through a bounded, monotone squashing nonlinearity—a modified tanh-like activation function—that maps the result back to the system-standard $\left[-1,1\right]\;$ range. The exact parameterization of the persistence term and activation function is proprietary, but the structure of the update rule follows a synchronous “current inputs plus a small carryover from t−1 → nonlinear squashing” structure applied uniformly to all concepts. At the system level, heterogeneity in aiHumanoid behavior therefore arises from differences in the underlying causal graph and edge weights and subject initialization states rather than from concept-specific update functions. The exact proprietary coefficients are not disclosed, but the functional sequence is uniform across the model.

In total, 7 clinically relevant pediatric ALL genotypic configurations were selected on the basis of known biological and prognostic associations: ETV6::RUNX1, ETV6::RUNX1 + FHx, ETV6::RUNX1 + Ikaros Del, ETV6::RUNX1 + PAX5 Del, ETV6::RUNX1 + radiation exposure, ETV6::RUNX1 + TEL LOH, and ETV6::RUNX1 + p16 Del.

Each genotype-specific simulation cohort comprised 25 virtual subjects (*n* = 175 total), spanning 6 developmental age strata (0, 2 to 3, 5, 10, 15, and 20 years). Intersubject variability was introduced through controlled perturbations of baseline concept states within predefined genotype- and age-specific ranges, rather than stochastic sampling from an explicit probability distribution. Virtual subjects represent internally consistent instantiations of the same causal model structure, configured to reflect age- and genotype-dependent biological variation through controlled differences in baseline concept states and disease-context overlays rather than independent clinical observations sampled from a natural population. All subjects were initialized at a remission baseline state (T0) and simulated longitudinally through 2 post-remission checkpoints: T1 (3 months) and T2 (6 months). Simulation inputs were standardized to the system-wide −1 to +1 scale used for LCM modeling to ensure comparability across genotypes, ages, and outcome domains. In the present study, intersubject variability arose primarily from structured initialization and subgroup-specific biological configuration; no unrestricted iteration-level stochastic noise process was introduced unless explicitly stated.

The cohort size of 25 virtual subjects per genotype was selected as a structured design parameter for longitudinal trajectory exploration rather than through formal statistical power calculation and may be varied in future sensitivity analyses.

A summary of the outcomes monitored in this study is presented in Table 1.

**Table 1.** Outcome domains monitored at each simulated time point. Genotype- and age-stratified simulation design and outcome domain structure used for longitudinal evaluation of relapse-relevant system behavior within the aiHumanoid causal modeling framework. All categories shown represent simulation constructs and are not intended to reflect clinical risk stratification.

1.	Immune activation → The stimulation of immune cells to recognize and respond to threats, including leukemic cells.
2.	ALL_clonal evolution → A model-level representation of progressive leukemic diversification and selection pressure toward more aggressive or therapy-resistant subclonal states.
3.	ALL_development → The biological process through which normal progenitor cells transform into malignant lymphoblasts in acute lymphoblastic leukemia [1,4].
4.	ALL_facilitating the immune response to ALL → Mechanisms or interventions that enhance immune detection and destruction of ALL cells.
5.	ALL_inhibiting the immune response to ALL → Processes by which leukemic cells suppress or evade immune surveillance, promoting disease persistence or relapse.
6.	ALL_minimal residual disease (BC-ALL) → A model-integrated representation of residual post-treatment leukemic burden in B cell ALL, conceptually aligned with clinically measurable MRD but functioning within the network as a disease-state indicator rather than as a direct laboratory assay.
7.	ALL_pre-leukemic clone expansion → The growth of early abnormal cell populations with leukemic potential prior to full malignant transformation.
8.	ALL_relapse → The reappearance or progression of leukemia after a period of remission, typically due to treatment-resistant clones.
9.	ALL_relapse pressure index → A composite model summary intended to quantify the overall longitudinal momentum or pressure toward relapse relevant system states.
10.	ALL_relapse risk score → A summary metric characterizing the relative burden of relapse-associated biological pressure within a given subject or genotype configuration, derived from key model indicators such as HLE, MRD, and clonal shifts.
11.	ALL_treatment resistance → The failure of leukemic cells to respond to chemotherapy or immunotherapy, often due to genetic mutations or protective microenvironments.
12.	QoL-physical wellbeing (PW) → A system-level state variable representing the physical health status and ability to perform daily activities, used as a proxy for treatment burden and functional wellbeing.

Quality of life (QoL)-physical wellbeing (PW) remained largely invariant across the present simulation intervals and was retained primarily as a system-stability reference variable rather than as a primary relapse-discrimination metric.

To summarize multidimensional relapse-relevant behavior within the simulation, 2 composite system-level state variables were implemented as standalone concepts within the LCM: the ALL_relapse risk score and the ALL_RPI. These metrics function as normalized aggregations of coordinated biological activity across multiple outcome domains and are intended to characterize relative system states and temporal momentum rather than to estimate absolute clinical probabilities or replace established relapse markers. The ALL_relapse risk score provides a time point-specific summary of relapse-associated biological activity, while the RPI captures longitudinal escalation or persistence of relapse-relevant dynamics across post-remission intervals. Both metrics are drawn from weighted inputs related to clonal evolution, MRD, treatment resistance, and immune modulation [7,10,11], with detailed structure, functional behavior, and operational definitions provided in Appendix 2 (Table 2).

**Table 2.** External concordance of key simulated pediatric ALL behavior patterns. External concordance table summarizing qualitative and directional alignment between key simulated pediatric ALL behavior patterns and established features of relapse biology reported in the literature [8–10]. These comparisons are intended as biological and clinical anchoring rather than as patient-level validation, calibrated prediction, or reproduction of absolute relapse frequencies.

Modeled trajectory pattern	Interpretation within the simulation	External anchor in pediatric ALL literature	Type of concordance	Scope of claim
Mid-childhood developmental windows show heightened relapse-relevant activity	Developmental state modulates relapse-related system sensitivity	Age-dependent developmental differences in pediatric ALL biology and relapse-associated processes are qualitatively described in the relapse literature [8–10]	Directional/qualitative	Biological plausibility only
ETV6::RUNX1-only profiles remain relatively stable over time	Favorable-risk genotype context is associated with lower relapse-oriented system divergence	Favorable-risk ETV6::RUNX1 behavior is qualitatively consistent with established pediatric ALL patterns [8–10]	Directional/qualitative	Not calibrated to clinical event rates
Minimal residual disease emerges as a dominant contributor to relapse-oriented system change	Residual post-treatment leukemic burden functions as a major relapse-associated state variable	MRD is a well-recognized relapse-associated clinical and biologic feature in pediatric ALL [8–10]	Mechanistic/qualitative	Not equivalent to laboratory MRD quantification
Clonal evolution contributes prominently to longitudinal relapse divergence	Progressive subclonal selection and diversification drive relapse-oriented system momentum	Clonal evolution is widely implicated in pediatric ALL relapse biology and often precedes overt recurrence [8,9]	Mechanistic/temporal	Not a phylogenetic reconstruction
Treatment resistance is a major component of modeled relapse pressure	Resistant biology contributes to persistence and recurrence within the simulated system	Treatment resistance is a central feature of pediatric ALL relapse progression [8–10]	Mechanistic/qualitative	Internal model alignment only
Relapse-relevant divergence emerges progressively rather than abruptly	Biologic relapse pressure accumulates longitudinally before overt relapse-like system states	Empirical relapse studies indicate that MRD and clonal evolution can precede overt hematologic recurrence [8,9]	Temporal/directional	Not a direct estimate of relapse timing
Relative ordering of relapse-associated processes is preserved	MRD, clonal evolution, and treatment resistance dominate before overt relapse-like system states	The literature supports this ordering as a plausible sequence in relapse development [8–10]	Rank-order/qualitative	Hypothesis-generating only

### Statistical characterization of trajectory dynamics

To summarize trajectory behavior and characterize modeled differences between simulated cohorts, we used a descriptive nonparametric framework centered on the HLE of median difference and Cliff’s delta effect size. These measures provide complementary assessments of magnitude, directionality, and ordinal separation of within-model trajectory differences without reliance on distributional assumptions. HLE-based change-from-baseline profiles were used to summarize longitudinal differences between predefined simulation checkpoints (T0, T1, and T2) and should not be interpreted as continuous-time biological trajectories.

Longitudinal change within the simulated system was evaluated by computing paired differences between time points (T1–T0, T2–T0, and T2–T1) for each virtual subject and summarizing these differences using the HLE as a measure of typical within-subject change. Cliff’s delta was used to quantify directional dominance and the strength of separation between simulated trajectory states.

Because virtual subjects were generated through deterministic, controlled perturbations of baseline concept states rather than stochastic sampling from a defined population distribution, all statistical summaries were interpreted descriptively rather than inferentially. No population-level *P* value interpretation, hypothesis testing, or statistical significance framework was applied.

To support structured identification of coordinated trajectory changes, a descriptive thresholding approach was used. Outcome domains were flagged when both a material Hodges–Lehmann shift (|HLE| > 0.1) and a large ordinal effect size (|Cliff’s delta| ≥ 0.474) were observed. These thresholds were used solely as internal pattern-detection criteria within the simulation and do not represent clinical decision thresholds or inferential statistical tests.

Effect size and directionality were summarized using Cliff’s delta [14], providing a distribution-free measure of the relative strength and direction of change across virtual subjects. A Cliff’s delta threshold of |δ| ≥ 0.474 was used to indicate large effect, chosen to minimize false-positive relapse signals in a high-dimensional longitudinal virtual study.

Subject-level magnitude of change was summarized using the HLE [15], calculated as the median of all pairwise differences between paired time points (T1–T0, T2–T0, and T2–T1) for each outcome and subject, as an estimate of typical per-subject change. Together, these complementary statistical measures were used to identify coherent longitudinal patterns within the simulation rather than to estimate external statistical power, clinical performance, or generalizability.

All relapse “risk” metrics in this study represent relative biological propensity within the simulated system, not absolute clinical probability. All results were normalized to the system-standard −1 to +1 scale, and SST methodology was used as an internal structured review process for concept definitions, causal directionality, and output plausibility (Appendix 1), rather than as an external validation procedure. While the aiHumanoid simulation platform is proprietary, the model logic relevant to this study—including outcome domain definitions, composite metric construction, update-rule structure, statistical thresholds, and interpretive criteria—has been fully specified to support independent conceptual replication and methodological scrutiny without requiring access to source code.

## Results

### Exemplar application: Pediatric ALL relapse

This section reports the longitudinal behavior of relapse-relevant biological domains within the simulated pediatric ALL system across post-remission intervals. Results are presented as patterns of within-system change detected across genotype and developmental age strata, using paired comparisons between remission baseline (T0) and post-remission checkpoints (T1 and T2). Longitudinal shifts were characterized using the HLE to summarize subject-level change, and Cliff’s delta to quantify directional dominance at the cohort level. These results describe relative system behavior and temporal evolution within the simulation, rather than clinical outcome frequencies or patient-level risk predictions.

Importantly, all references to risk in this section denote relative, simulated risk as defined in the Methods section, reflecting comparative patterns of relapse-relevant biological activity within the aiHumanoid framework rather than estimates of clinical event probability or clinical decision guidance.

### T1 versus T0: Early relapse-relevant trajectory changes following remission in pediatric virtual ALL

This analysis examines early post-remission longitudinal change within the simulated pediatric ALL system by comparing remission baseline (T0) with the first post-remission checkpoint (T1, 3 months). Longitudinal behavior was evaluated across 175 virtual subjects stratified by 7 ETV6::RUNX1-based genotypic profiles and 6 developmental age cohorts (0, 2 to 3, 5, 10, 15, and 20 years). Changes across 12 relapse-relevant biological domains were characterized using the HLE to summarize subject-level shifts, Cliff’s delta to quantify directional dominance at the cohort level, and predefined descriptive trajectory criteria to identify coordinated within-system change relative to baseline (T0). A risk-flagging criterion was applied as an internal pattern-detection heuristic to identify coordinated domain shifts within the simulation, rather than to infer clinical relapse probability. Table 3 and Table S1 summarize the distribution of HLE values across outcome domains and age strata for the T1 versus T0 comparison.

**Table 3.** Summary of early relapse-associated patterns (T1 versus T0). Summary of genotype-specific relapse-associated patterns observed during the early post-remission interval (T1 versus T0). Dominant domains represent the principal contributors to divergence from remission baseline within the simulation. Developmental windows indicate ages at which relapse-associated biological activity was most prominent. Results describe relative system behavior within the aiHumanoid framework and do not represent clinical relapse probabilities.

Genotype	Peak developmental window	Dominant relapse-associated domains	Longitudinal pattern
ETV6::RUNX1	No consistent age-specific peak	None consistently elevated	Stable post-remission profile with minimal behavior divergence
ETV6::RUNX1 + FHx	No consistent age-specific peak	None consistently elevated	Similar to baseline genotype with limited longitudinal change
ETV6::RUNX1 + p16 deletion	5–10 years	Minimal residual disease, clonal evolution	Age-dependent relapse-associated activity confined primarily to mid-childhood
ETV6::RUNX1 + IKZF1 deletion	5–10 years	Minimal residual disease, clonal evolution	Intermediate behavior divergence with temporally restricted activation
ETV6::RUNX1 + PAX5 deletion	5–10 years (peak at 10 years)	Minimal residual disease, clonal evolution, treatment resistance	Persistent multi-domain relapse-associated behavior activation beginning in mid-childhood
ETV6::RUNX1 + radiation exposure	10–15 years (peak at 15 years)	Treatment resistance, clonal evolution	Sustained relapse-pressure dynamics extending from mid-childhood into early adulthood
ETV6::RUNX1 + TEL LOH	Mid-childhood	Clonal evolution, relapse pressure index	Persistent relapse-associated behavior divergence intermediate between baseline and high-risk genotypes

### Genotype-linked patterns of early relapse

Among the 7 genotypes modeled, ETV6::RUNX1 + PAX5 deletion and ETV6::RUNX1 + radiation exposure showed the most prominent early relapse-associated trajectory divergence within the simulation.

Throughout this section, “flagged” denotes satisfaction of predefined descriptive trajectory criteria and does not imply statistical significance, discrete biological activation, or a clinical relapse event.

The ETV6::RUNX1 + PAX5 deletion genotype demonstrated one of the strongest early relapse-associated trajectory profiles within the simulation, with 369 flagged domain events identified across all developmental age strata. The most prominent activity occurred at age 10 years, where 9 of 12 relapse-relevant domains satisfied the predefined descriptive trajectory criteria. Notable contributors included clonal evolution (HLE = +0.22, Cliff’s *d* = 0.54) and minimal residual disease (MRD) (HLE = +0.18, Cliff’s *d* = 0.49). Collectively, these findings indicate sustained early relapse-associated trajectory activation beginning in mid-childhood within the modeled system.

The ETV6::RUNX1 + radiation exposure genotype produced the highest overall burden of relapse-associated trajectory activity, with 379 threshold-satisfying domain shifts observed across the simulation. Prominent signals were evident at age 15 years, where treatment resistance (HLE = +0.19, Cliff’s *d* = 0.51) and clonal evolution demonstrated substantial trajectory divergence from baseline. These coordinated changes were sustained across multiple developmental stages and were associated with persistent relapse-pressure dynamics extending from mid-childhood into early adulthood. Intermediate-risk genotypes such as ETV6::RUNX1 + p16 deletion and ETV6::RUNX1 + Ikaros deletion showed limited and age-dependent relapse activity. In contrast, ETV6::RUNX1 alone and ETV6::RUNX1 + FHx (family history) showed minimal biological deviation from remission, with consistently low HLE and Cliff’s *d* values across all ages.

**Fig. 1.**
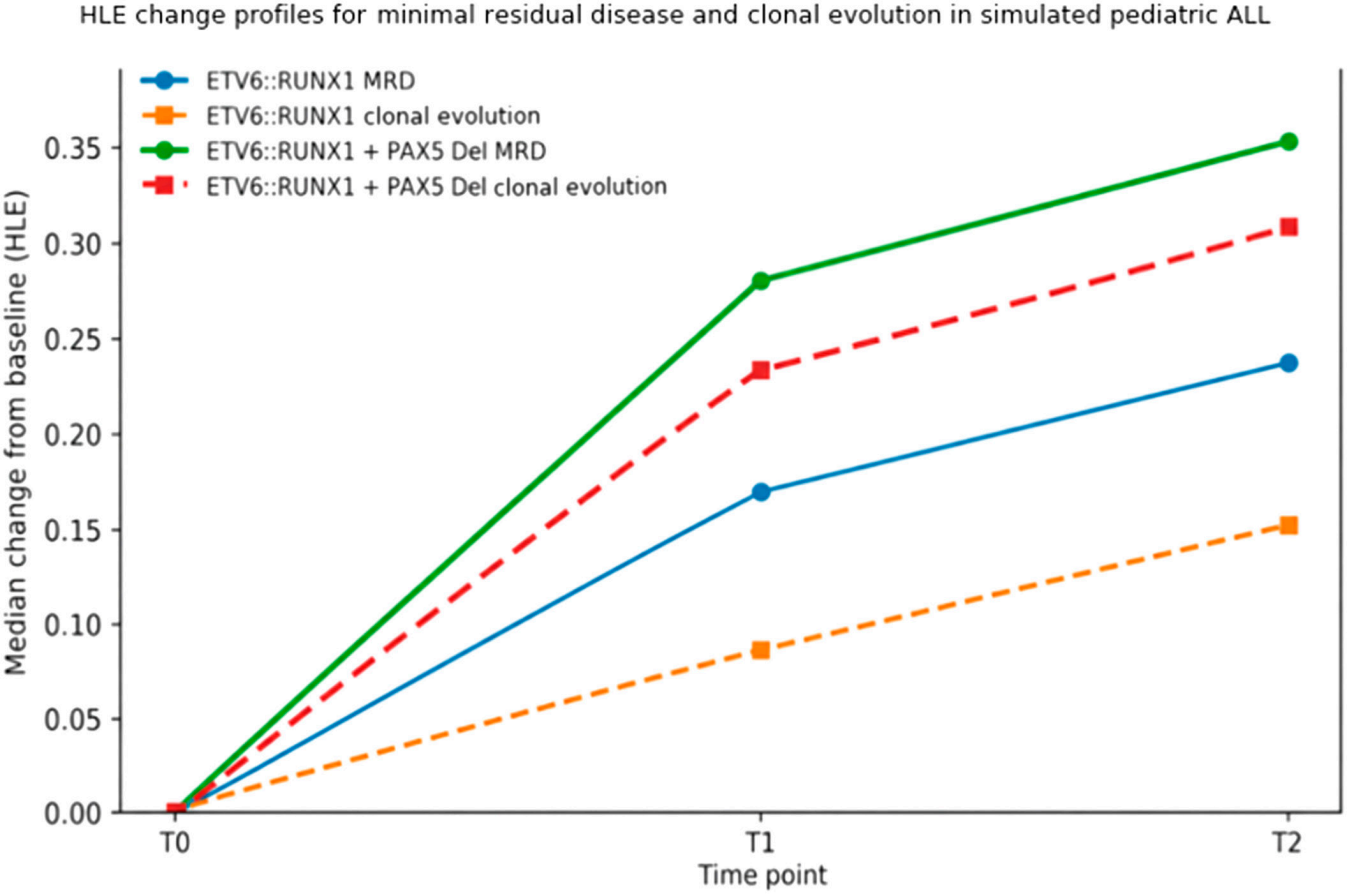
Longitudinal HLE profiles for MRD and clonal evolution in simulated pediatric ALL. Values represent Hodges–Lehmann estimates of median change from remission baseline (T0 = 0) for ETV6::RUNX1 baseline and ETV6::RUNX1 + PAX5 deletion cohorts across T1 and T2. These profiles summarize the magnitude and direction of change relative to baseline within the simulation and do not represent absolute concept activation states or continuous biological trajectories.

These longitudinal changes are summarized using HLE-based change-from-baseline trajectories, highlighting the differential progression of relapse-associated biological activity between ETV6::RUNX1 baseline and ETV6::RUNX1 + PAX5 deletion cohorts (Fig. 1).

### T2 versus T0: Relapse-relevant trajectory changes at 6 months post-remission in pediatric ALL

To investigate the potential for early subclinical relapse, we analyzed virtual longitudinal profiles comparing T2 (6 months post-remission) with T0 (remission baseline) across pediatric ALL subjects [3,4]. This comparison was conducted using 2 complementary statistical approaches: HLE [15] for subject-level shifts, and Cliff’s delta [14] for study population-level effect sizes. The HLE estimates for T2 versus T0 across the 12 relapse domains and 6 age cohorts are summarized in Table 4 and Table S2.

**Table 4.** Summary of relapse-associated system behavior at T2 versus T0. Summary of genotype-specific relapse-associated system behavior for the T2 versus T0 comparison. Values represent maximum Hodges–Lehmann estimator values observed across age strata for each genotype. Results describe relative simulated system behavior and do not represent clinical relapse probabilities.

Genotype	Peak age by RPI	Max RPI	Max risk score	Max MRD	Max clonal evolution	Max treatment resistance	Main pattern
ETV6::RUNX1	20	0.113	0.233	0.258	0.155	0.034	Low activity
ETV6::RUNX1 + FHx	20	0.123	0.238	0.253	0.159	0.036	Low activity
ETV6::RUNX1 + Radiation	15	0.360	0.361	0.234	0.214	0.254	Intermediate activity
ETV6::RUNX1 + TEL LOH	0	0.434	0.406	0.244	0.234	0.316	Elevated activity
ETV6::RUNX1 + p16 Del	0	0.443	0.426	0.243	0.246	0.318	Elevated activity
ETV6::RUNX1 + IKZF1 Del	0	0.492	0.473	0.299	0.286	0.345	High activity
ETV6::RUNX1 + PAX5 Del	15	0.575	0.554	0.365	0.330	0.377	Highest activity

RPI, relapse pressure index; MRD, minimal residual disease

We defined simulated high-risk relapse strata as those that met the following predefined descriptive trajectory criteria: |HLE| > 0.1 and |Cliff’s delta [14]| ≥ 0.474. This approach identifies methodologically consistent and biologically interpretable relapse-associated signals within the simulation.

Out of 72 possible age × outcome combinations (6 age cohorts × 12 outcome domains), 18 met the predefined high-risk descriptive trajectory criteria (25%). The most pronounced risk signals were seen in the 2- to 3-year and 5-year age cohorts, which together accounted for 11 of the 18 flagged combinations.

Developmental age strongly influenced relapse-associated trajectory behavior within the simulation. The 2- to 3-year and 5-year cohorts exhibited the greatest burden of coordinated relapse-associated activity and together accounted for 11 of the 18 age-by-domain combinations meeting the predefined descriptive trajectory criteria. The most prominent contributors were clonal evolution, MRD, the RPI, and treatment resistance. The 10-year cohort demonstrated a more moderate pattern of trajectory divergence, whereas the youngest (age 0) and oldest (age 20) cohorts remained comparatively stable throughout the simulation interval.

Among the 12 relapse-related domains evaluated, 4 emerged as particularly influential contributors to relapse-associated trajectory behavior. Clonal evolution was repeatedly identified across ages 2 to 3 through 10 years, frequently exceeding HLE values of +0.14 and Cliff’s delta values above 0.50. MRD demonstrated persistent elevation, particularly in the age-5 cohort (HLE = +0.230, Cliff’s *d* = 0.51). Treatment resistance was repeatedly identified between ages 5 and 15 years, suggesting the emergence of therapy-resistant biological states within the simulation. RPI also increased across the 2- to 3-year through 10-year cohorts, indicating accumulation of coordinated relapse-associated biological activity over time.

Illustrative high-risk stratum: At age 5, MRD showed a subject-level increase (HLE = +0.23) and a large effect-size estimate Cliff’s *d* = 0.51, fulfilling the predefined high-risk trajectory criteria.

**Fig. 2.**
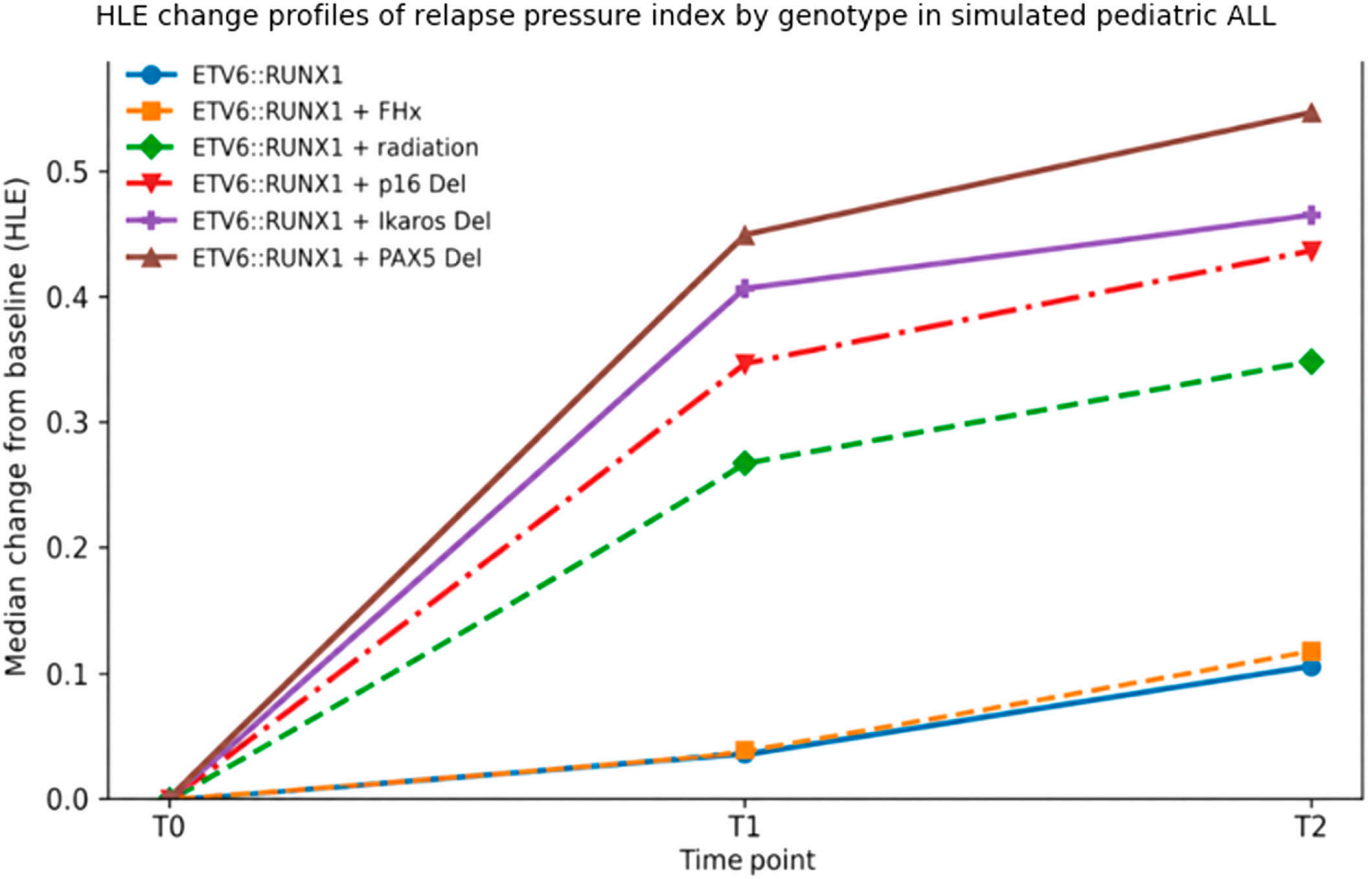
Longitudinal HLE profiles of RPI change by genotype in simulated pediatric ALL. Values represent Hodges–Lehmann estimates of median change from remission baseline (T0 = 0) for ETV6::RUNX1 baseline, ETV6::RUNX1 + family history (FHx), and mutation-associated genotypes across T1 and T2. These profiles summarize longitudinal changes in the RPI relative to baseline and do not represent absolute concept activation states or continuous biological trajectories.

Genotype-specific divergence in relapse-pressure dynamics is summarized using HLE-based change-from-baseline trajectories, demonstrating progressive separation between ETV6::RUNX1 baseline, ETV6::RUNX1 + FHx, and mutation-associated genotypes over time (Fig. 2).

### T2 versus T1: Later relapse risk signals between 3 and 6 months post-remission

To assess continued relapse risk trajectories after initial post-remission surveillance, we compared virtual pediatric ALL profiles between T2 (6 months post-remission) and T1 (3 months post-remission) across 175 subjects. Using HLE [15] and Cliff’s delta [14], we characterized relapse-relevant changes in terms of subject-level magnitude and effect-size separation. We defined high-risk strata as those with |HLE| > 0.1 and |Cliff’s delta| ≥ 0.474.

Out of 72 age × domain strata, 21 met the predefined descriptive trajectory criteria (29.2%), indicating an increased burden of biological recurrence activity in this interval compared to earlier comparisons. The 2- to 3-year, 5-year, and 10-year age cohorts were especially vulnerable, contributing 16 of the 21 high-risk strata (76%).

Age-stratified analysis demonstrated that the 2- to 3-year, 5-year, and 10-year cohorts accounted for 16 of the 21 age-by-domain combinations satisfying the predefined descriptive trajectory criteria. The 2- to 3-year cohort exhibited continued increases in clonal evolution, MRD, and treatment resistance, including MRD values of HLE = +0.19 and Cliff’s *d* = 0.50. The 5-year cohort demonstrated prominent RPI and treatment resistance signals, whereas the 10-year cohort showed renewed divergence in clonal evolution and treatment resistance, suggesting accumulation of relapse-associated biological burden over time. In contrast, the 15- and 20-year cohorts displayed relative stabilization, with only 2 domains meeting the descriptive trajectory criteria across both age groups.

Clonal evolution, MRD, and treatment resistance remained the most informative domains during the T2 versus T1 interval. Clonal evolution demonstrated persistent activity across the 2- to 3-year through 10-year cohorts, frequently exceeding Cliff’s delta values of 0.50 and HLE values above +0.15. MRD remained particularly prominent in the 2- to 3-year and 5-year groups, supporting the persistence of residual leukemic activity within the simulated system. Treatment resistance continued to emerge across intermediate developmental stages, suggesting progressive adaptation toward therapy-resistant biological states. A more subtle increase in the ALL_relapse risk score was also observed among older children between ages 10 and 15 years, although not all predefined descriptive trajectory criteria were satisfied.

One notable addition was subtle emergence of ALL_relapse risk score in older children (age 10 to 15), with HLE > +0.11 and Cliff’s *d* > 0.45, though not reaching all predefined criteria thresholds.

### High-risk stratum example

At age 2 to 3, MRD was again prominently elevated (HLE = +0.19, Cliff’s *d* = 0.50), meeting the predefined descriptive trajectory thresholds and reinforcing its role as a core recurrence-associated driver.

Summary: Simulated stratification of relapse-associated trajectory patterns in pediatric ALL

Analysis across the 3 post-remission intervals (T0 versus T1, T0 versus T2, and T2 versus T1) identified consistent genotype- and age-associated patterns of relapse-relevant trajectory divergence within the simulated pediatric ALL system. Classification of simulated strata was based on the number of outcome domains satisfying the predefined descriptive trajectory criteria, the magnitude and directional consistency of longitudinal change (HLE > 0.1 and |Cliff’s delta| ≥ 0.474), and the persistence of these patterns across multiple time intervals.

Among the 7 genotypes evaluated, ETV6::RUNX1 + PAX5 deletion and ETV6::RUNX1 + radiation exposure consistently exhibited the strongest relapse-associated trajectory behavior. The ETV6::RUNX1 + PAX5 deletion genotype demonstrated persistent activation of MRD, clonal evolution, and treatment resistance beginning in mid-childhood, with as many as 9 of 12 monitored domains satisfying the predefined descriptive trajectory criteria at age 10 years. Similarly, the ETV6::RUNX1 + radiation exposure genotype produced the greatest overall burden of flagged domains and displayed sustained trajectory divergence in clonal evolution and treatment resistance from ages 10 to 15 years. Within the simulation, these genotypes demonstrated the most persistent and coordinated relapse-associated biological activity throughout the post-remission period.

Intermediate patterns of relapse-associated activity were observed in the ETV6::RUNX1 + p16 deletion and ETV6::RUNX1 + IKZF1 deletion genotypes. These profiles demonstrated age-dependent and temporally restricted activation of relapse-relevant domains, most commonly between ages 5 and 10 years. Although IKZF1 deletion demonstrated higher peak values for several individual composite measures, radiation exposure produced broader and more persistent multi-domain activation across developmental stages and observation intervals.

In contrast, the ETV6::RUNX1 baseline genotype and the ETV6::RUNX1 + family history (FHx) genotype remained comparatively stable throughout the simulation, with no domains consistently satisfying the predefined descriptive trajectory criteria and only minimal HLE and Cliff’s delta shifts observed across post-remission intervals.

Developmental age emerged as an important modifier of simulated relapse-associated trajectory behavior. The age 2- to 3-, 5-, and 10-year cohorts consistently demonstrated the greatest burden of coordinated activity across domains related to MRD, clonal evolution, and treatment resistance, particularly within the higher-risk genotypic configurations. By comparison, infants (age 0) and older adolescents (age 20) exhibited substantially lower levels of trajectory divergence across all evaluated genotypes. These findings support the hypothesis that developmental state may influence relapse-associated biological dynamics within the modeled system and suggest that mid-childhood may represent a period of increased longitudinal sensitivity to relapse-relevant perturbations in genotype-defined simulation strata.

This developmental signal gradient supports hypothesis generation around age-stratified relapse-associated biology, particularly during mid-childhood in genotype-defined simulation strata, while recognizing that these findings arise from a mechanistic simulation framework rather than direct clinical outcome data.

## Discussion

The present study provides a pediatric ALL-focused proof of concept for studying disease trajectories using LCMs. Rather than focusing solely on prediction at isolated time points, the approach models disease progression as an evolving causal system and summarizes trajectory behavior using descriptive nonparametric measures of magnitude and directional separation within the simulated system. While pediatric ALL relapse served as a biologically well-characterized exemplar in this work, the current manuscript should be interpreted as demonstrating architectural and analytic feasibility within a single disease exemplar rather than establishing validated disease-agnostic generalizability.

These findings highlight a fundamental limitation of current relapse risk stratification approaches in childhood ALL, particularly those relying on static or time point-dependent measurements such as MRD. While MRD remains a powerful clinical tool for quantifying residual leukemic burden, it does not fully capture the underlying biological processes that drive relapse, including clonal evolution, treatment resistance, and immune modulation. As a result, relapse-associated biological activity is often inferred from discrete measurements rather than modeled as a continuous biological trajectory. Recent advances in MRD assessment, including next-generation sequencing-based approaches and protocol-driven risk stratification strategies, further emphasize both the strengths and inherent limitations of MRD as a time point-dependent biomarker, particularly in its ability to reflect evolving disease dynamics over time [18–20]. In contrast, the aiHumanoid framework models relapse as a dynamic and evolving process, enabling the identification of temporal patterns and biologically meaningful transitions that are not readily captured by conventional MRD-based approaches.

The methodological contribution of this work lies in the combined application of Cliff’s delta [14] and the HLE [15] to longitudinal childhood ALL simulation outputs. Together, these complementary measures were used to summarize coherent within-model subject- and cohort-level state transitions within the simulated system. In particular, HLE provided a summary of early subclinical domain-level change, supporting the characterization of sustained longitudinal shifts that may precede overt phenotypic divergence within the modeled relapse framework. In the present study, these measures are best interpreted as within-model descriptors of longitudinal contrast, magnitude, and directional consistency rather than as conventional population-level inferential statistics.

### Genotype- and age-modulated longitudinal relapse patterns

A central observation arising from this analysis is the emergence of distinct, genotype-associated longitudinal relapse patterns within the simulated system, modulated by developmental age. Rather than forming static risk categories, genotypes clustered according to the persistence, coordination, and temporal extent of relapse-relevant domain activation across post-remission intervals.

### Genotypes exhibiting sustained, multi-domain longitudinal activation

ETV6::RUNX1 + PAX5 deletion and ETV6::RUNX1 + radiation exposure showed consistent divergence from baseline system equilibrium across all evaluated time points, with up to 9 of 12 outcome domains concurrently activated in mid-childhood age strata. Within the simulation, these profiles reflect persistent system-level momentum, indicating heightened sensitivity to relapse-relevant biological perturbation across developmental stages.

### Genotypes characterized by transient or age-restricted activation

ETV6::RUNX1 + IKZF1 deletion and ETV6::RUNX1 + p16 deletion displayed relapse-relevant domain engagement that was temporally constrained, most prominently between ages 5 to 10. Within the simulated system, these patterns suggest developmental windows during which genotype-specific biological effects exert a greater influence on longitudinal system behavior.

### Genotypes demonstrating longitudinal stability

ETV6::RUNX1 alone and ETV6::RUNX1 + family history remained near baseline equilibrium throughout the post-remission simulation, with minimal Hodges–Lehmann shifts and consistently nondominant directional effects. These profiles illustrate relative system stability within the modeled relapse framework across ages and time points.

### Developmentally modulated longitudinal sensitivity patterns

A prominent feature of the simulated relapse dynamics was the emergence of developmentally modulated longitudinal sensitivity patterns that were reproducible across genotypes. Within the simulation, the developmental stages corresponding to ages 2 to 3, 5, and 10 years were consistently associated with higher concurrent activation across relapse-relevant domains, whereas infancy (age 0) and late adolescence (age 20) remained comparatively stable. These age-associated patterns were observed independent of genotype, suggesting that developmental state exerts a system-level influence on longitudinal relapse-related behavior.

These developmental inflection points may reflect the convergence of multiple biological processes, including immune reconstitution, bone marrow microenvironmental remodeling, and age-dependent therapy responsiveness. Visualization of longitudinal trends (Tables 3 and 4) further illustrates that domains related to MRD, clonal evolution, and treatment resistance emerge as dominant contributors to coordinated system-level change during mid-childhood [8–10]. Importantly, these patterns should be interpreted relative shifts in simulated biological activity rather than as direct indicators of clinical relapse timing or probability, or patient-level prediction.

In contrast to digital disease modeling approaches including digital twin frameworks in medicine and oncology [21–26], which often emphasize individualized prediction or correlative machine learning representations, the aiHumanoid platform employed here is structured as a causal, physiologically grounded, and dynamically adaptive system. Disease progression is represented as an evolving network of interacting biological processes, enabling characterization of longitudinal momentum, temporal escalation, and counterfactual trajectories within the modeled system. By capturing trajectory-level dynamics across both genotype and developmental stage, this framework emphasizes time-aware system behavior rather than point-based risk estimation. In this sense, the present work should be read as an architectural proof of concept in pediatric ALL rather than as a claim of superiority over alternative trajectory-modeling approaches.

### Simulation metrics and methodological relevance

The composite system-level indices derived in this study—the ALL_relapse score and the RPI—proved useful for summarizing coordinated longitudinal behavior across multiple relapse-relevant biological domains within the simulation. When interpreted alongside primary contributors such as MRD and clonal evolution, these metrics provide a structured means of characterizing temporal momentum and persistence of relapse-associated activity. Importantly, these indices are intended as internal descriptors of system state within a causal simulation framework rather than as direct estimators of clinical relapse probability or substitutes for established clinical markers.

Several pediatric ALL concepts used in the present LCM are intentionally modeled at an aggregated biologic or systems level rather than at the granularity of single molecular assays or cell-state hierarchies. For this reason, variables such as MRD, clonal evolution, immune facilitation/inhibition, and the relapse composite indices should be interpreted as biologically grounded model constructs whose role may be mechanistic, proxy-like, or integrative depending on network position. Table S3 was therefore added to clarify how these concepts relate to known pediatric ALL biology while also making explicit the abstraction limits of the current exemplar.

### Future applications

The LCM trajectory framework described here has not been validated outside of the current pediatric ALL exemplar. However, the underlying modeling strategy could be adaptable to other diseases characterized by nonlinear progression or episodic relapse, including autoimmune disorders, neurodegenerative diseases, solid tumor recurrence, and treatment response dynamics in chronic conditions. Such adaptations should be regarded as future applications requiring their own biological calibration, methodological specification, and empirical validation rather than as conclusions established by the current manuscript. Future work will therefore focus primarily on strengthening external anchoring and validation within defined disease settings before broader cross-disease extension is inferred.

### Limitations and future integration

This work is grounded in human physiology-informed virtual simulations and is therefore subject to the constraints inherent to any model-based analysis. The findings presented here should be interpreted as characterizations of simulated longitudinal system behavior rather than as validated clinical outcomes. Future efforts will focus on integrating empirical longitudinal datasets to calibrate simulation-derived trajectories against observed relapse patterns, as well as extending the framework to explore adaptive therapeutic strategies and immune-modulated disease dynamics within the same causal structure.

Because model structure, causal relationships, and edge-weight assignments were partially informed by published pediatric ALL biology, the qualitative concordance analysis presented here does not constitute a fully independent external validation procedure and may contain an element of literature-informed circularity.

As longitudinal pediatric relapse datasets continue to mature and federated data-sharing initiatives expand, opportunities for prospective alignment between simulated trajectories and real-world outcomes are expected to increase. Such integration would allow further evaluation of how trajectory-based modeling may complement existing empirical approaches without replacing established clinical decision frameworks.

### Anchoring of simulation outputs to established clinical and biological patterns in pediatric ALL

Although the present study does not attempt patient-level validation or clinical prediction, the longitudinal simulation outputs were evaluated for concordance with established qualitative patterns reported in pediatric ALL relapse literature. Specifically, the emergence of mid-childhood developmental windows characterized by heightened relapse-relevant activity, the relative biological stability of favorable-risk ETV6::RUNX1-only profiles, and the prominence of MRD, clonal evolution, and treatment resistance as dominant contributors to relapse dynamics are all qualitatively consistent with features described in real-world pediatric ALL progression [8–10].

The observation that relapse-relevant system divergence arises early and evolves longitudinally rather than appearing abruptly is also consistent with empirical studies indicating that biological relapse processes often precede overt clinical recurrence. These trajectory-level patterns are directionally aligned with contemporary relapse literature showing that clonal evolution and MRD dynamics can precede overt hematologic recurrence in pediatric ALL cohorts [8,9]. Importantly, the present framework does not seek to reproduce absolute relapse frequencies, patient-level timelines, or calibrated clinical event probabilities. Instead, it is anchored by reproducing the relative ordering, temporal structure, and directional behavior of relapse-associated biological processes observed in pediatric ALL cohorts. This form of qualitative and rank-order anchoring supports the interpretation that the simulated trajectories remain biologically constrained while preserving the framework’s intended role as a hypothesis-generating and methodological tool. These relationships are summarized in Table 2.

### Reproducibility statement

The aiHumanoid simulation engine is proprietary, and its source code is not publicly disclosed. However, all causal structures, biological domains, composite metric definitions, update rules, thresholds, and statistical evaluation criteria required for independent conceptual replication are fully specified within this manuscript. As a result, the methodological framework and analytical logic can be independently implemented and evaluated using alternative causal or mechanistic simulation platforms without access to the underlying aiHumanoid codebase. The present study therefore does not provide computational reproducibility in the open-source sense, but it does aim to provide sufficient methodological transparency for conceptual replication and critical evaluation.

## Conclusion

This longitudinal simulation study supports the feasibility of combining Cliff’s delta [14] and the HLE [15] to characterize genotype- and age-modulated relapse-relevant trajectories within a causal modeling framework for pediatric ALL. Rather than identifying clinical risk categories, the results illustrate how coordinated system-level patterns can emerge early in the post-remission period and evolve across developmental stages within a controlled computational environment.

By integrating causal simulation with nonparametric change-detection metrics, this approach provides a scalable methodological framework for exploring relapse dynamics, evaluating the biological impact of emerging genomic variants, and generating testable hypotheses for future longitudinal studies. More broadly, it illustrates how longitudinal, trajectory-focused modeling can extend beyond static risk scoring toward deeper mechanistic understanding of disease evolution within a simulated environment.

LCMs may provide a useful computational approach for exploring disease trajectories as evolving causal systems. By combining concept-level modeling with statistical trajectory analysis, the LCM framework may become a flexible tool for studying complex disease dynamics across a wide range of biomedical applications. However, in the context of the present study, that claim is limited to the pediatric ALL proof-of-concept exemplar. Additional biological benchmarking, methodological expansion, and empirical validation will be required before stronger claims regarding clinical performance or cross-disease generalizability can be justified.

## Ethical Approval

Not applicable. This study was conducted entirely in silico using population-based virtual pediatric ALL models. No human participants, patient-level clinical data, or identifiable biological specimens were involved; therefore, formal ethics approval and informed consent were not required.

## Data Availability

The datasets generated and/or analyzed during the current study (simulation outputs and longitudinal trajectories) are available from the corresponding author on reasonable request. The underlying implementation of the aiHumanoid engine is proprietary and not publicly released.

## Appendix 1

Sober second thought (SST) methodology

To ensure the internal reliability, coherence, and physiological realism of all simulation outputs, we employed the sober second thought (SST) methodology. SST is an embedded expert-guided validation framework developed specifically for use within the aiHumanoid [13] simulation platform. It operates as a second-layer logical audit that independently evaluates the plausibility, directionality, and system-level consistency of all defined concepts, weights, and causal relationships prior to result presentation to the user

SST does not assess predictive accuracy or validate outcomes against external data. It does enforce structural and logical constraints by verifying that modeled relationships are internally coherent, physiologically interpretable, and aligned with the specific analytic objectives defined for each simulation run. This process prevents unsupported inference, contradictory causal propagation, or reasoning drift within the modeled system.

Each concept node—whether physiological, pathological, or composite—must pass 3 tiers of evaluation under SST:

Causal coherence check:

Verifies that all direct inputs and outputs are logically and biologically consistent. For example, increases in minimal residual disease must propagate appropriately to relapse risk but not illogically to unrelated domains.

Directional weight audit:

Confirms that weight signs (positive or negative) reflect known or expected real-world relationships. For example, treatment resistance must positively influence ALL_relapse risk score and RPI but not suppress them.

Dynamic feedback stability:

Assesses each node for participation in feedback loops and attractor cycles to ensure that no unintentional oscillatory or runaway behavior occurs without cause.

Validation of SST for this study

The SST process was validated in this simulation through:

Cross-genotype consistency checks: All 7 ALL genotypes were tested to confirm reproducible behavior of core relapse domains under identical baseline inputs.

Edge perturbation testing: System stability and directional logic were tested by temporarily adjusting weights (±0.1 to ±1.0) for key inputs (e.g., clonal evolution → risk score) to confirm predictable system-level responses.

Empirical alignment: The SST-verified network outputs were compared against known clinical relapse patterns (e.g., PAX5 Del and Radiation showing highest risk; ETV6::RUNX1 baseline showing lowest) for real-world face validity.

Together, these SST validations ensure that the LCM-based simulation behaves in a biologically interpretable manner allowing biologically interpretable evaluation of composite relapse scores and temporal pressure indices.

## Appendix 2: Composite metric definitions and implementation

To quantify emergent simulation relapse dynamics in pediatric ALL, we implemented 2 system-level composite metrics in aiHumanoid [13] V11.0: the ALL_relapse risk score and the ALL_RPI. Both are defined as standalone LCM nodes with direct inputs from relapse-relevant outcome domains and are normalized to the standard –1 to +1 LCM scale. While they share overlapping inputs, they serve distinct analytic functions—one as a time point-specific estimator of relapse burden, and the other as a temporal momentum indicator.

All concept weights, input domains, and output behaviors were verified using the sober second thought (SST) methodology to ensure biological coherence and simulation stability.

1. ALL_relapse risk score

Purpose:

A time point-specific estimator of relative relapse burden at T0, T1, or T2. It aggregates relapse-relevant input domains into a single scalar output reflecting current risk.

Direct inputs:

• ALL_clonal evolution
• ALL_minimal residual disease (BC-ALL)
• ALL_treatment resistance
• ALL_pre-leukemic clone expansion
• ALL_inhibiting the immune response to ALL

Behavior:

• Weighted sum of domain activity at a given time point.
• Normalized to the –1 to +1 scale.
• Used to stratify simulated subjects according to relative relapse-associated biological burden within the model.

Operational calculation:

Calculated as the weighted mean of domain shifts exceeding the prespecified effect-size contrast threshold (HLE > ±0.1), with rank-based directional summaries and Cliff’s delta [14] used to summarize directional consistency, magnitude, and contrast strength.

2. ALL_relapse pressure index (RPI)

Purpose:

A temporal momentum metric that captures the system’s dynamic biological pressure toward relapse over time (T0 → T2). It detects sustained system behavior-based risk even in the absence of threshold crossing.

Direct inputs:

• ALL_clonal evolution
• ALL_minimal residual disease (BC-ALL)
• ALL_treatment resistance
• ALL_development
• ALL_inhibiting the immune response to ALL

Behavior:

• Emphasizes temporal persistence, escalation, and slope of input trends.
• Weights are applied dynamically based on direction, rate, and duration of change.
• Particularly sensitive to sustained upward HLE values and increasing Cliff’s delta across intervals.

Operational calculation:

Computed by aggregating system behavior slopes across T0, T1, and T2 for relapse-relevant domains. Domains with persistent increases contribute more heavily to the RPI value.

Summary:

Together, these composite metrics form the core analytic backbone for estimating the simulated relapse-associated system behavior within the aiHumanoid framework [13]. The risk score identifies subjects at elevated risk at a given time, while the RPI detects evolving systemic momentum toward relapse—enabling both snapshot characterization of simulated system state and trend-aware evaluation of relapse-associated activity.

## Appendix 3

Composite metric definitions for longitudinal relapse analysis

To distill complex, multidimensional relapse behavior into clinically interpretable summaries, we derived 2 composite metrics from the 12 relapse-related outcome domains:

• ALL_relapse score: A normalized summary index reflecting the cumulative relapse burden for each subject or genotype. This score was calculated as the weighted mean of domain changes meeting the prespecified within-model contrast criteria (HLE > ±0.1) and Cliff’s delta (|*d*| > 0.474) to summarize contrast magnitude and directionality.
• Relapse pressure index (RPI): A dynamic systems-level metric capturing the temporal stress exerted by relapse-prone biological pathways. RPI was computed by aggregating the system behavior slopes of domains showing sustained upward HLE trends across T0, T1, and T2, emphasizing MRD, clonal evolution, and treatment resistance.

Both composite indices were normalized to the standard −1 to +1 LCM scale and verified using the sober second thought (SST) methodology for internal consistency. These metrics were then used to categorize system relapse risk across age–genotype strata and help guide interpretability in longitudinal analyses.
